# The many boar identities: understanding difference and change in the geographies of European wild boar management

**DOI:** 10.1080/09640568.2023.2269312

**Published:** 2023-11-03

**Authors:** Erica von Essen, Kieran O’Mahony, Marianna Szczygielska, Thorsten Gieser, Virginie Vaté, Aníbal Arregui, Ludek Broz

**Affiliations:** a Høgskolen i Innlandet, Faculty of Applied Ecology, Elverum, Norway; b Czech Academy of Sciences, Institute of Ethnology, Prague, Czech Republic; c Societies, Religions, Secularisations, French National Centre for Scientific Research, Paris, France; d Social Anthropology, Universitat de Barcelona, Barcelona, Spain

**Keywords:** biopolitics, necropolitics, hunting, biosecurity, wildlife management

## Abstract

Wildlife management across Europe is increasingly characterised by a ‘war on wild boar’. In response to epidemiological and economic threats to pig production and agriculture, state agencies, policymakers and hunting organizations have altered their management as they attempt to contain wild boar. Through a cross-section overview of eight European countries with differentiated strategies – the Czech Republic, France, Germany, Great Britain, Norway, Poland, Spain, and Sweden – we analyze five critical components of contemporary wild boar management: categorizing, responsibilizing, calculating, controlling, and sanitizing. We consider three critical triggers that change how wild boar and, by extension, a range of other ‘wild’ species are managed in relation to the aforementioned categories: (over)abundance and population growth, biosecurity crises, and technological innovation. While these triggers, on one hand, might streamline transborder management policies, we show how wild boar also uproot longstanding wildlife management cultures by transforming hunting traditions, landowner-hunter relations and meat handling practices.

## 1. Introduction

The lives and deaths of wild boar (*Sus scrofa*) have been significantly influenced by their interaction with humans, as well as by various social, political, and ecological factors that are present in their respective habitats. Their resurgence across Europe since WWII has challenged some long-established co-existence and wildlife management approaches (Fleischman 2017; Palencia *et al*. 2023a). Over this period, positive responses of wild boar to socio-environmental changes, including rural depopulation, afforestation, rewilding, agricultural intensification, urban expansion, and climate change, have enabled their populations to grow at rapid rates (Vetter *et al*. 2020). Furthermore, they now reside not only in forests and farmland, but also in towns and cities (Arregui 2023; Marin 2023; Oelke, Müller, and Miggelbrink 2022), and regions where they have returned (Hearn, Watkins, and Balzaretti 2014; O’Mahony 2022).

As wild boar move across different physical, political, and moral boundaries, the strategies for managing them have become increasingly disputed by different interest groups (Grady *et al*. 2019). Growing concerns about disease transmission and agricultural disturbance have caused social-political conflicts at local, national, and supranational levels (Fulgione and Buglione 2022). These changes have been further complicated by critical events, such as the worldwide epidemic of African Swine Fever (ASF), which has prompted authorities and various publics to reconsider how they wish to handle wild boar (Broz, Arregui, and O’Mahony 2021; EFSA AHAW Panel 2014; Acevedo *et al*. 2022). Wild boar, whose presence and risk have been highly politicised, are now considered something of a battlefield where laws and regulations on management are continuously changing (Vajas *et al*. 2023; von Essen 2020). Therefore, critical questions arise regarding the appearance of management and whose responsibility it is to solve the wild boar ‘problem’ (Keuling, Strauß, and Siebert 2016).

Given this context, our paper presents a change-focused summary of wild boar management in various European countries. We explore how policy frames wild boar and how the species is managed through socio-legal *assemblages*, which consist of multiple actors, hunting practices and monitoring technologies. Furthermore, we examine how wild boar have become agents of change and prompted broader advancements in wildlife management. We start with a literature review, summarizing relevant research on wildlife politics, management and governance. Specifically, we draw on literature that addresses the exercise of power in wildlife management, regarding both its bio- and necropolitical aspects (Biermann and Anderson 2017; Braverman 2015). The subsequent section details our methods and the countries we studied: the Czech Republic, France, Germany, Great Britain, Norway, Poland, Spain, and Sweden. Later, we present the significant characteristics of wild boar management, and compare and contrast the case countries we studied. This is organized around five key components of management addressing how: (i) wild boar are categorized; (ii) responsibility is administered; (iii) populations are monitored; (iv) management is practiced; and (v) meat is regulated. Based on these components, the final discussion analyses three interconnected causes which fundamentally alter wild boar management – (over)abundance and population growth, biosecurity crises, and technological innovations – and their drivers, and how this could potentially impact the management of wildlife in general.

This paper advances earlier research that broadly dealt with ungulate management in Europe (Putman 2011) by concentrating on wild boar. We provide details of contemporary management approaches, including the participation of hunters, and present potential future scenarios. Although recent research has summarized the diverse legal frameworks related to wild pigs in the US (Smith *et al*. 2023), it is especially crucial to address the European scenario due to its intricate political environment. Clarifying cross-border contrasts and similarities can assist managers and sectors who aim to coordinate wild boar management at various complex levels, specifically in response to ASF risk (Acevedo *et al*. 2022; Skjerve *et al*. 2018). Finally, considering the ongoing cull-based tactics utilized in wild boar management, the paper contributes to empirical scholarship that not only emphasizes necropolitical aspects of wildlife management, that is, schemes focused on hunting and culling, but also demonstrates how non-human species can challenge, modify or disrupt these schemes and frameworks.

## 2. Literature review

### 2.1. Governing political territory

Wildlife management emerges through complex social, legal, political and ecological landscapes (Ojalammi and Blomley 2015; Chrulew and Wadiwel 2016). The socio-legal assemblages that comprise management – inscribed through discourses, legislative texts, institutions, places, technologies and material practices – are the outcomes of historic regional approaches intersecting with transnational governance frameworks (Braverman 2015). This has been highlighted by Putman (2011) who identified four regionally distinct approaches to ungulate management in Europe – Central European (Germanic), Scandinavian, Southern European and Anglo-Saxon – which differ according to the extent governments intervene in the (i) lives and deaths of wildlife, and (ii) coordinate management. The demarcation of territories and spatial-legal conceptions of ownership, property and responsibility connect governments with publics and individuals, and are critical to how wildlife management is organised across Europe (Blomley 2016).

Within and across territories, wildlife management has increasingly been theorised through two interrelated political regimes, bio- and necropolitics. Biopolitics is a modern rationality which foregrounds life and populations as its subject (Biermann and Mansfield 2014; Biermann and Anderson 2017; Lorimer 2015), seeking to “ensure, sustain and multiply life…to put [it] in order” (Foucault 1978, 138). While biopolitical logics might enable death (or its threat), they commonly exercise a (bio)power which promotes the life of *desirable* species, populations and individuals. In contrast, necro-political regimes foreground death and/or killing as primary modes of managing and regulating *undesirable* life. Death is a form of (necro)power, an “active political process[es]…necessary for the maintenance of other kinds of life” (Margulies 2019, 152). Key to the enactment of wildlife (bio/necro)politics are i) the categorization of non-human life, and ii) the governmentalities that shape responsibility for management.

### 2.2. Governing through categorization

Categories, classifications and labels are fundamental to wildlife management as they emplace value and establish boundaries between the ab/normal, un/desirable and im/pure (Biermann and Mansfield 2014; Wolfe 2015). Such systems commonly (i) order animals, (ii) frame their belonging, and (iii) spatialize their relations relative to anthropocentric conceptions of territory and place. Primarily, non-humans are categorized as *wild, domestic* or *feral*, distinctions historically framed by legal and moral conceptions of ownership, property, responsibility and non-human autonomy (Florence 2014; Donaldson and Kymlicka 2011; Braverman 2015). In European law, animals are generally classified as (1) under somebody’s care and ownership (*res propria*); (2) belonging to no-one (*res nullius)*; (3) belonging to the state (*res communis*); or (4) as feral (once belonging to someone but no longer under their control). These relational framings of ownership intersect with legal categories zoning animals according to human utility, for example, whether they are identified as pets, game, agricultural animals, zoological exhibits, or pests (Philo and Wilbert 2000). Such categories commonly determine *who* should be responsible for their lives and deaths and, importantly, *how*.

In addition to ownership and property, spatial-ecological logics also organize species’ belonging through conceptions of non/native-ness (O’Mahony 2020; Warren 2007). This is often analogous to moral notions of a species’, population’s or individual animal’s (un)naturalness (Lavau 2011). These may coalesce with the notion of ‘invasiveness’, applied to proliferate populations perceived as a threat to ecological, agricultural and economic security (Barker *et al.* 2013; Simberloff 2013). Such categories, critical to the shaping of animals’ moral and legal geographies, are frequently bound up in broader political projects relating to nationhood and territory (Gibbs, Atchison, and Macfarlane 2015; Keil 2023).

Wildlife whose mobilities commonly cross property, administrative, legal and moral borders can problematize these ordering logics (Snijders 2012). In some cases, animals traversing adjacent territories might simultaneously be identified as a *native game* species, or persecuted as a *non-native, invasive pest* (Milton 2011). Urban wildlife, for example, is often labelled ‘out-of-place’ and commonly does not have the same *de jure* or *de facto* status as their ‘wild’ conspecifics (Ikeda *et al*. 2019) and, typically, becomes the responsibility of different types of wildlife managers (von Essen and Redmalm 2023; Oelke, Müller, and Miggelbrink 2022).

### 2.3. Governing through responsibilization

Wildlife management in Europe is commonly spread through multi-level social-political arrangements connecting states and governments to individual citizens, communities, and publics (Rose and Miller 1992). These arrangements identify *who* and *how* such actors should be involved; they shape conduct, and diffuse social responsibility for, in the context of wildlife management, protection, monitoring, control, or sanitation in wildlife management (Crowley, Hinchliffe, and McDonald 2018; Emond, Breda, and Denayer 2021; Hinchliffe and Lavau 2013; Maye *et al*. 2014). Importantly, such arrangements are not necessarily determined by top-down disciplinary means but rely upon willing actors to “produce the ends of government by fulfilling themselves rather than being merely obedient” (Rose, O’Malley, and Valverde 2006, 89). In other words, effective management relies on public enrolment, particularly that of hunting communities.

Wildlife management is founded in long-standing practices integral to social relations, such as monitoring and hunting. According to modern political rationalities, however, they might also be understood as ‘technologies of government’. These, following Miller and Rose (2008), can often be separated into two interrelated forms, knowledge production and intervention. As an enactment of the former, monitoring and surveying enable various authorities to formalize the presence of wildlife within particular territories and address them as populations (Biermann and Mansfield 2014). Practices which count individuals, estimate populations and predict trends become critical means through which wildlife are governed by states, communities and individuals (Boonman-Berson, Driessen, and Turnhout 2019). As well as population distribution, density and presence, contemporary monitoring increasingly encompasses wildlife disease and health (Hinchliffe and Lavau 2013).

Monitoring commonly informs practices of control and is dependent on how wildlife itself is categorized. Censuses and surveys compiled by farmers, landowners, hunters, game management units, or government agencies might inform practical interventions, such as cull quotas. These may differ in intention, from biopolitical rationales seeking to sustain and protect- for example, through supplementary feeding and habitat conservation-, to necropolitical agendas which cull or eradicate. These might involve variants of killing, such as recreational hunting and culling interventions (Crowley, Hinchliffe, and McDonald 2018), and are enacted through practices- such as trapping, shooting or poisoning- which vary according to socio-legal contexts. Likewise, responsibility for control varies, but is frequently delegated among actors with differing degrees of independence and power. For example, it may be delegated to individuals or local hunting teams who report to regional hunting associations, who themselves are overseen by state authorities (Putman 2011; Vajas *et al*. 2023).

Informed by this body of literature, this paper not only considers how wild boar management differs across Europe, but also how these regimes might be transformed by social, political, ecological or epidemiological circumstances (Broz, Arregui, and O’Mahony 2021; Emond, Breda, and Denayer 2021; Hinchliffe and Lavau 2013).

## 3. Method

This paper has emerged from a collaborative project exploring how disease risk and biosecurity is altering wild boar-human relations across contemporary Europe.^1^ We follow the general contours of eight national regimes- the Czech Republic, France, Germany, Great Britain, Norway, Poland, Spain, and Sweden- and consider how they: (i) categorize; (ii) responsibilize; (iii) calculate; (iv) control; and (v) sanitize wild boar presence.^2^ Analysis was ordered around these components as they appear critical to (bio/necro)political strategies of wildlife management. Altogether, these countries exemplify diverse models, re-iterations, and adaptations of wildlife management. Moreover, they may be understood to carry their own wild boar *risk portfolios*, understood as the assessment of the threat posed by wild boar based on their diverging ecological histories, social-political settings, and cultural practices.

**Figure 1.**
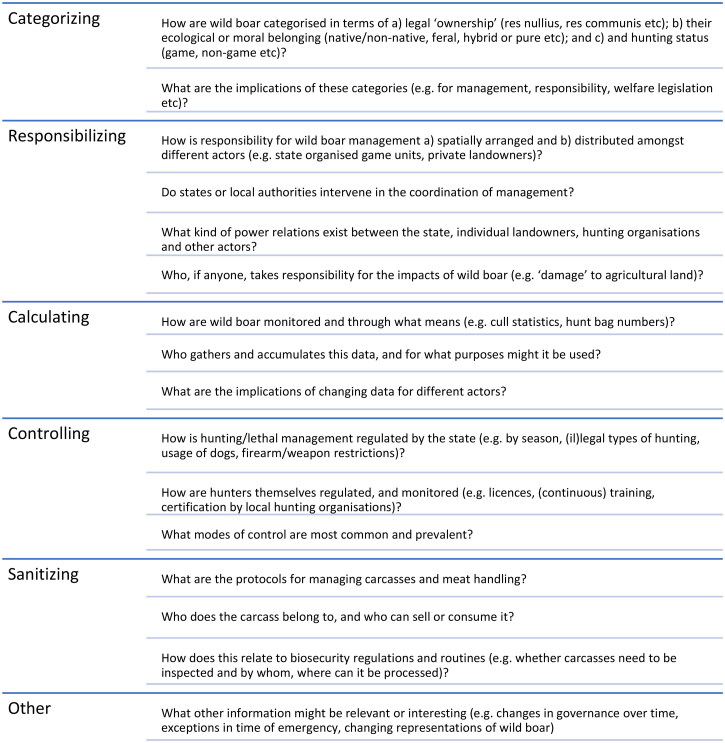
Question prompts for analysis.

To gather relevant information, the two lead authors von Essen and O’Mahony provided question prompts relating to the different components (i–v) to their co-authors to help summarise management in their country of expertise (e.g. see Figure 1). The co-authors have all been conducting long-term research on wild boar in these countries, and their responses were based on primary knowledge and the collation of secondary data, such as policy documents. Finally, the lead authors synthesized the findings from all countries. While co-authors were not specifically asked about change, this was a powerful theme that emerged. In the following sections, we analyze the key features of wild boar management, drawing out their similarities and differences. In the final discussion, we highlight some key triggers of change and consider their relevance beyond wild boar management.

## 4. Wild boar management frameworks

### 4.1. Categorizing

A fundamental category of wildlife management is ‘native-ness’ and wild boar management in Europe is framed by their historic ‘native range’ throughout the continent (Keuling, Strauß, and Siebert 2016). European countries with wild boar today have commonly followed one of two historical trajectories: continuous wild boar presence, or extirpation and recolonization. Developing this understanding, our analysis highlights how (i) convoluted these trajectories have sometimes been; (ii) different categories and logics override one another; and (iii) belonging is geographically, morally and ecologically contingent.

Although legislation in the Czech Republic, France, Germany, Poland, Spain and Sweden reflects wild boar’s ‘native’ status, this has been historically problematized by fluctuating populations and abundance in these countries. For example, in the Czech Republic wild boar were considered de facto outlaws between 1786 and 1947, during which they were nearly extirpated from the ‘open’ landscape and managed only in game preserves. By contrast, in Poland a period where wild boar were perceived as de facto ‘invasive’ occurred at the beginning of the twenty first century, having recolonized following extirpation in the 1950s. Similarly, while never fully extirpated, a very low population post-WWII in Germany rapidly proliferated in the latter half of the twentieth century, notably during the 1980s, and was referred to as the ‘Wildschwein Plage’ (wild boar plague) (Fleischman 2020). These recent trends mirror population developments across Europe (Keuling, Strauß, and Siebert 2016). Importantly, we found that native range is not merely conceived as a horizontal spread, but might also be altitudinal. For example, in regions of the Czech Republic, France, and Poland, wild boar above a certain altitude are sometimes perceived as beyond their native range.

In national territories where wild boar were wholly, as opposed to partly, extirpated and have since recolonized, categorization diverges. In Sweden, for example, wild boar were once widely present (around 5000 years ago) before disappearing in the 1600s. Following recolonization in the twentieth century – largely after releases from hunting enclosures –, until 1987 they were considered invasive, after which Swedish parliament (1986/87:JoU15) recategorized them as native. In contrast, wild boar in Norway- a population spawning from migration across the Swedish border in the last decade- have *not* been reassimilated. Rather, they appear on the national invasive species list (previously called ‘black list’) and are labelled an invasive species estimated to have a ‘very high impact’ on nature and societal interests (Gederaas, Moen, and Skjelseth 2012), despite archaeology highlighting their presence until around 1000 AD (Rosvold *et al*. 2010).

In Great Britain, wild boar (re)introduction has similarly led to distinct policy responses between nations. Although unclear, wild boar likely disappeared from the island sometime between the 13th and 16th Centuries (Yamamoto 2017). As contemporary ‘wild’ populations originate from a growth in wild boar farming in the 1980s, in England they have been legislatively classified as ‘feral wild boar’ and ‘animals no longer normally present’ (Infrastructure Act 2015). In contrast, in Scotland they are understood as a ‘formally native’ animal ‘outwith its native range’ and, due to doubts surrounding genetic purity, categorized as ‘feral pigs’ (NatureScot 2022). Indeed, in many national contexts, native-ness pertains to conceptions of *genetic purity* rather than geographic origin. Wild boar in regions of Spain may be classified as invasive on account of crossbreeding with domestic pigs, such as feral Vietnamese potbellied pigs, and are referred to as *cerdolí* (Delibes-Mateos and Delibes 2013). Similarly, in France wild boar illegally released from game reserves in the 1970s–1990s have concerned authorities due to their uncertain genetic purity (Gigounoux 2017), and have been labelled ‘cochonglier’ or, less often, ‘sanglichon’ (mixing ‘sanglier’ (wild boar) and ‘cochon’ (pig)).

These categories become fundamental components of socio-legal management frameworks, effectively helping to construct *what* wild boar *are* and, as we shall consider, how they are meant to live or die. Interestingly, in many European countries their belonging – understood through native-ness, for example – has rarely been consistent. Extirpation, recolonization, reintroduction or naturalization continuously define legislative status.

### 4.2. Responsibilizing

Responsibility for wild boar and their management depends on several socio-legal factors, and the ways in which power is defused through multi-actor governance arrangements. In our analysis, three aspects of responsibility emerged as significant, relating to how (i) wild boar become property; (ii) their ecologies become political-economic matters; and (iii) multi-actor management is formalized. First, in almost all the countries, wild boar are legally categorized as *res nullius*, a category commonly applied to game animals meaning they belong to no one until captured or killed (Cavanagh 2018). Upon death they often become the property of the hunter or landowner, *res propria,* although this varies nationally.

For example, in Spain (similar to France) wild boar belong to the person whose action *first* injured (or killed) an animal (Ley de Caza 1970, Art 22/2). In contrast, in the Czech Republic this rule customarily identifies who was the successful shooter while legally, the hunted boar belongs to the ‘hunting ground user’ (Zákon č. 449/2001 Sb. § 2). In Poland, ownership is dependent on land agreements: wild boar killed by hunting ground tenants become their property, whereas on unleased land they belong to ground managers (Karpiuk 2019). An ‘appropriation right’ for wild boar exists in Germany, and while connected to land ownership, it can be sold to users. Great Britain is a subtle outlier to other systems, for while wild ungulates (and other wildlife) are also understood to be *res nullius*, this is not formally the case with wild boar which are categorized ‘feral’ as opposed to ‘wild’, inferring the underlying premise that they *were* once someone’s property. However, practice follows standard UK shooting legislation and stalkers need permission from landowners to shoot on their land, with ownership of carcasses contingent on specific agreements.

States tend to delegate responsibility for management of wild boar to private actors, whether landowners, hunting associations or individuals. As part of such governance arrangements; however, state influence varies, as does the extent to which national management is systematically coordinated systematically (Putman 2011). In Germany, according to federal hunting law (*Bundesjagdgesetz BJagdG*) the ‘right of appropriation’ means hunting ground managers and/or users have a ‘duty for population regulation’ (*Pflicht zur Hege*), making management a compulsory and personal responsibility.

One can discern a kind of ‘hunters-pay-principle’ in some central and southern European clusters. In the Czech Republic, hunting users are responsible for ‘damage’ caused by game animals within those grounds, but not on neighboring land. In France, the ‘Federation of Hunters’ must present the objectives of their hunting plans six years in advance, pay compensation for wild boar damage, and maintain an inventory of such payments (Domas-Descos 2012; Gigounoux 2017). Likewise, a ‘hunters-pay-principle’ is present in Spain, where damage costs are the responsibility of hunting tenants or, if there is no provable negligence, landowners; and similarly in Poland, where managers of hunting districts, rather than landowners, are responsible for wild boar damage, then the Chief Forester, and eventually the state treasury. Responsibility is thus spread *de facto* between hunting organizations and the State Forest’s National Forest Holding.

Enacting a somewhat less tightly governed system to those above, with no penalties for unmet quotas or damages, in Sweden, landowners have autonomy to manage wild boar as they wish. They may lease rights to hunters- often the case with farmers-, sharing a partial commonality to the German ‘appropriation right’. The Swedish Environmental Protection Agency also permits targeted wild boar culls (*skyddsjak*t) in special circumstances of foreseen damage on public owned land, when there is a risk of traffic collisions, an ASF outbreak, or wild boar entering gardens. In Great Britain, government policy is the least intrusive- unless on state-owned Forest- and management decisions are devolved to individual landowners and communities. As such, nobody bears responsibility for damage and there is no system of compensation, although not unlike in Sweden, recent amendments to law have given the government the possibility of intervening on private land through ‘control orders’ (Infrastructure Act 2015).

To meet cull quotas and to mitigate damages, higher wild boar numbers have partly prompted shifts in the ease of obtaining licenses for hunters. Countries differ wildly in the intensity, time duration and supervision required (Putman 2011: own observations). In several places, however, one can observe a move toward more readily obtainable licenses that do not require years of training or additional qualifications. ln Sweden, for example, ‘quick’ tests now grant licenses after an intensive weekend of training followed by a practical and written exam, which is now standardized and administered digitally. Such courses have been criticized for enabling people to ‘buy’ licenses at the cost of adequate mentorship and cultural foundation but are also seen as necessary to deal with wild boar (Tickle, von Essen, and Fischer 2022).

Quick exams can also be found in countries with more rigorous processes, such as in Mecklenburg-Vorpommern in Germany, where intensive ‘manager training courses’ offered by private hunting schools can qualify hunters in fifteen days, although subsequent mentorship is usually expected (*Jägerprüfungsverordnung-JägerPVO M-V*). Similarly, French hunting associations and government intend to make hunting more accessible in response to growing wild boar populations and declining hunter numbers. However, rather than simplifying learning, increasing accessibility implies reducing costs, in particular licenses that enable hunting throughout the country, as opposed to single regions and the tax fees when registering for hunting permits. Such changes might enhance the findings of recent studies that indicate wild boar are increasingly a ‘first port’ for many young hunters (Tickle, von Essen, and Fischer 2022).

Although the legal categorization of wild boar (as *res nullius*) is similar across countries, responsibility is governed diversely. Conceptions of property and ownership vary, as does the liability to control both populations and their movements. We hypothesize that this also affects how one views the wild boar – as a resource or pest. Importantly, we can also see a trend whereby governments are easing some restrictions, such as in the case of firearms and the entry requirements to becoming a hunter, in response to the changing presence and challenges of wild boar management. This, in turn, may go on to change hunting cultures, demographically and socially.

### 4.3. Monitoring

Related to responsibility, monitoring is also diversely overseen and administered by national authorities, both in relation to the different actors involved, and the methods used. When it comes to disease monitoring, surveillance is either passive or active. Countries place different weight on passive and active depending whether they are in an active epidemic state or not. Cardoso *et al*. (2022) declare that the present challenge is both the lack of integration between passive and active surveillance, and failure to harmonize disease monitoring with appropriate population size monitoring. It is often the latter that proves difficult. Due to their abundance and elusiveness, estimations in many countries- such as France, Germany, Poland and Sweden- rely on the aggregation of ‘harvesting’ data (in other words, animals killed) or ‘hunting bags’, outtakes which are calculated as percentages of a population. Typically, countries adhering to Germanic-derived systems of governance require more detailed reports, such as including non-hunting related mortalities, such as the Czech Republic, Sweden and Norway. Sweden and France are highlighted by Cardoso *et al*. (2022) as examples of more rigorous national passive surveillance that also draws on citizen science reporting, such as SVA (https://rapporteravilt.sva.se/Home/Inledning) and SAGIR (Desvaux *et al*. 2021).

In contrast to these systems where the gathering of statistics across whole political territories is compulsory, in Great Britain there is a lack of systematic monitoring, and individual landowners are neither obliged to report data on presence nor numbers killed. Instead, estimations may be based on arbitrary and ad-hoc reports, outdated figures, or else speculative computer models based on limited samples.

In countries with more top-down regulation, obligatory reports flow along different hierarchical relations. In some situations, hunting bags are self-reported directly to managing authorities, either *via* reports or databases directly while elsewhere – such as in Poland, and Spain – bag numbers are first gathered by hunters and synthesized across a whole region, before being sent to authorities. In Germany, for example, hunters report to the *Untere Jagdbehörde*, who compiles hunting bags for the state hunting agency (*Obere Jagdbehörde*). In other contexts, namely, the Czech Republic, a combination of these approaches is used. Here, all hunting ground users conduct game censuses at the county level and subsequently submit this data to the state hunting administration. However, hunters must also submit monthly reports on the fulfilment of game management plans that contain both monthly hunting bags – including sex and age – as well as annual figures (Zákon č. 449/2001 Sb., § 38).

Beyond monitoring based on hunting bags and cull data, in some countries and cases alternative methods are employed to count and estimate populations. For example, in Poland trial drives (*pędzenie próbne*) take place every February, wherein wild boar are chased across a limited terrain by beaters and head counted by trained observers. In contrast to these long-standing practices, elsewhere technological developments have given rise to new monitoring forms. For example, in the Forest of Dean, a state managed forest in England, forestry authorities conduct a thermal imaging census every March and estimate population through distance sampling (Gill 2022). Similarly, in Norway, the state performs periodic surveillance in locations where wild boar presence is suspected using drones with thermal vision, an effective method at detecting wild boar in forests but poor for estimating densities. These results, along with harvest data, observations logged onto the civilian platform Artsobservasjoner (‘Observations of species’) and ‘fallviltsregistret’– the systematic registration of wildlife traffic accidents – inform Norwegian estimates. Sweden has a similar system of collation.

Critical events or crises also transform the kinds of monitoring methods. For example, methods such as thermal imaging are not only used in locations with limited, (re)introduced populations (such as Great Britain and Norway), but increasingly during ASF outbreaks or when localized wild boar eradications are planned, such as along the Danish-German wild boar border fence (Sauter-Louis *et al*. 2022). Additionally, subsidies can become an important monitoring tool (as well as a control mechanism), as in the Czech Republic where bounties of 80 EUR have been given for wild boar shot above the five-year hunting bag average of hunting grounds during ASF outbreaks. In Norway, where the stated population goal is the “fewest possible wild boar, spread over the smallest possible area” (Miljødirektoratet 2019), subsidies are also given for reporting found wild boar to central authorities, despite no active or historical contagion of ASF in the country.

Going forward, ENETWILD, a European network of wildlife professionals estimating wild boar density and abundance (Vicente *et al*. 2019), now seek to harmonize ways of assessing population and distributions. The idea is that hunting bags should not only be submitted but authenticated by other actors and datasets. To this end, emerging databases such as the European Wildlife Observatory (https://wildlifeobservatory.org/) and Mammalnet, along with its tool Mammalia, seek to collate public observations of both living and dead wild boar. These initiatives, building also on The European project APHAEA (harmonized Approaches in monitoring wildlife Population Health, And Ecology and Abundance; https://www.aphaea.eu/), propose homogenized monitoring schemes for both wild boar populations and the spatial-temporalities of associated diseases. They also indicate a move towards the digitization of wildlife monitoring, an EU goal (Cardoso *et al*. 2022).

### 4.4. Controlling

While previous sections have addressed how responsibility for control might be diffused through various governance arrangements, here we consider ways in which wild boar management is practiced. Specifically, this includes (i) seasonality; (ii) hunting methods and modalities; and (iii) the role of dogs. A main point of contrast is open seasons vs year-round culling. Seasonality is still enacted in France. In the north, stalking can usually take place from June to September/October, and for drive hunts from November to February, allowing a closed season to protect lactating sows and piglets (Code de l‘Environnement, Livre IV, Titre II, Chapitre IV). These seasons vary according to region, for example, in the south of France, they may continue from June to March. In contrast, however, many other countries have either changed their policy (Czech Republic, Germany and Poland), or never enacted such policy (Great Britain, Sweden), meaning wild boar can be lethally controlled year-round. While no closed season exists in Norway, legislation prohibits the culling of sows with piglets, although piglets themselves are ‘fair game’ (*villtloven*). This legislative trend is, primarily, driven by increasing concerns about wild boar (overabundance) and their disease risks, and exemplifies a shift towards management as a form of necropolitics.

Historically, it has been normal to leave out food for wild boar, and in many countries a distinction is made between supplementary feeding and baiting. While the latter is still commonly permitted, increasingly the former is prohibited, further reflecting political desires to restrict rather than sustain populations (Schulz *et al*. 2019). In Norway and Sweden, legislation dictates that baiting sites must be strategically located away from highways or crops, and not distributed in concentrations higher than one per five-hundred hectares. Restrictions have also existed in Norway since 2020 which limit the maximal allowance of fodder (Hedmark *et al*. 2021). In Germany, baiting sites continue to be important – for example, accounting for approximately one third of the Lower Saxony hunting bag (Keuling, Strauß, and Siebert 2021) – and must be reported to the *Jagdbehörde*.

In the Czech Republic, Germany, Poland and Spain, wild boar are commonly hunted through a combination of methods: baiting, stalking, sitting or collective hunting (such as driven hunts). Driven hunts, or *battues*, are often understood to be the most effective method for regulating population, and are the primary method used in France (Vajas *et al*. 2023). While these are often limited by season in France, they may be allowed at other times when significant damage prompts targeted culling (such as in Spain). In contrast to most countries, drive hunting ungulates in Great Britain is illegal, so seated shooting or stalking are the principal methods, the latter commonplace on public land where management is politically contentious. Additionally, a recent development across Europe has been the approval and use of live-capture traps (Giménez-Anaya *et al*. 2020; Palencia *et al*. 2023a), particularly in urban-adjacent areas where firearm use is restricted (Giménez-Anaya *et al*. 2020). However, in countries where wild boar populations are sparse, such as Norway, live-capture traps have been relatively ineffective (Hedmark *et al*. 2021). The use of traps has also sparked some public and animal rights concern in Europe (Frank, Monaco, and Bath 2015; von Essen 2020), particularly when part of publicly visible culls, such as in urban Poland (Kowalewska 2019), Barcelona (Arregui 2023) or the Forest of Dean, England. Such opposition has prompted sabotage, problematizing the effectiveness of traps in management strategies.

Different killing methods- from hunting to culling- come with their own firearm and ammunition allowances. For example, in Poland hunting with full metal jacket firearms is outlawed and ammunition must be soft-pointed only (Regulation of the Minister of the Environment 2005, Journal of Laws 2005, No. 61 item 548 as amended). In the Czech Republic, shotguns with slugs can only be used for hunting piglets and yearlings in driven hunts, although during disease epidemic responses, this also applies to adult animals (Zákon č. 119/2002 Sb, 2014 amendment). Regulations on bullet energy also exist for regular hunting contexts, although not when culling trapped animals.

Whereas compound bows are not permitted everywhere in Europe, an exception is in Spain where the Ministry of Agriculture, Fisheries and Food has sanctioned special measures – such as trap captures, bowhunting and lethal veterinary interventions – *via* city councils. More broadly, an ever-broadening range of technologies have also transformed wild boar hunting, including silencers, thermal and infrared vision, specialized scopes, and various live-capture traps with smartphone alerts from sensors. These have spurred the legalization of night shooting, which in many contexts was banned and remains so for other game. In Sweden and Norway, for example, spotlighting was legalized in 2019 and 2021 respectively, contributing to a decreasing wild boar population in 2020–2021 (Hedmark *et al*. 2021).

A final issue of divergence across Europe regards dogs. In Great Britain, dogs are prohibited in the active hunting of ungulates, although they may be used to help locate or track injured or maimed animals. In most other countries, however, they are allowed, albeit with different restrictions. For example, in Poland dogs cannot be part of driven hunts, but solo hunters can use them when stalking wild boar (as opposed to deer). In the Czech Republic, dogs may be used in driven hunts, but must not be taller than 55 cm (Zákon č. 449/2001 Sb.§ 45). Dogs are also allowed for wild boar hunts in France and Germany. Unlike in France, in Germany, they are only meant to corner or flush wild boar, except in the latter where dogs are permitted to track wounded animals (typically dogs of the category Schweißhunde). In several countries, kennel associations focusing on ‘wild boar hunting dogs’ have become increasingly common as hunters have become more interested in investing in breeds such as the Slovakian *Kopov*, the Polish *Gończy*, and the Croatian *Posavski gonic*. The (mis)use of dogs for wild boar hunting has become contentious in some places, such as Sweden and Norway where criticism is frequently directed towards overly-aggressive central European wild boar dogs but which are, simultaneously, increasingly sought after for import (personal observations, 2023). In case of ASF outbreaks, dogs are typically prohibited in the ASF contagion area as they may exacerbate spread.

### 4.5. Sanitizing

A final, critical component of wild boar management is carcass and meat processing, with guidelines for rendering, butchering, handling and distribution commonly dependent on whether it is for private or commercial consumption. Sanitation- related to different biosecurity concerns- draws together a variety of actors, including hunters and veterinarians. Across Europe, current EU legislation (Regulation EC, No. 853/2004) requires hunters to be ‘trained persons’ who perform minor veterinary tasks in the field and deploy various biological knowledge, health diagnoses and welfare assessments (Emond, Breda, and Denayer 2021). Indeed, hunters increasingly seem to be acquiring ‘front line’ responsibilities to monitor, identify, report and contain pathogens and disease (Urner *et al*. 2020).

Primary among these are tests for *trichinella*, a parasite transmissible to humans through meat, which are diversely administered. Testing is compulsory throughout the EU if meat is for public consumption, but not necessarily for private consumption, as in France and Great Britain (Bison *et al*. 2022). Responsibility for testing, in these countries, is determined by the person who sells the meat, whether individual hunters licensed to trade or game dealers. In contrast, in the Czech Republic, negative samples mean carcasses may be (a) used by the hunting land users, (b) sold to game dealers for processing, (c) sold whole to private buyers, or (d) butchered in certified facilities and sold to customers. In Sweden, as part of a new initiative, hunters are now trained in meat handling are allowed to sell limited numbers of animals directly to consumers without using intermediary processing units, while in Norway meat can be sold if at least one hunter in a hunting team is certified to field-dress game (Hedmark *et al*. 2021).

Hunters are commonly trained or given guidance on how to take samples from carcasses, which are then sent to be tested by local (Germany, France) or national (Great Britain) veterinary agencies, although in other cases veterinarians might themselves collect samples from the field (Spain). *Trichinella* tests are mostly subsidized by governments, in some cases a recent development correlating with growing wild boar populations (Sweden) or regional ASF outbreaks (Germany). Diverging from other countries, Norwegian authorities pay hunters- 100EUR for shot males, 200EUR for shot females, and 300EUR for found carcasses or injured animals- to submit up to nine samples from a single animal, not only for *trichinella* testing but also other viruses and parasites, some of which are analyzed by the Swedish veterinary institute (SVA).

As highlighted by shifts to subsidize *trichinella* sampling, extraneous circumstances are also important in determining the governance of sanitary practices. For example, meat from regions contaminated by fallout after the Chernobyl disaster in 1986, including affected areas in the Czech Republic and Sweden, may have elevated radioactive caesium (CS-137) levels (in part, due to the consumption of mushrooms). Consequently, this is analyzed by authorities, with legal limits for human consumption set at 1500 bq/kg in Sweden, 600 bq/kg in the Czech Republic and 600 bq/kg in some German regions (Bundesamt für Strahlenschutz).

More significantly across the continent, however, are biosecurity protocols shaped by the emergence of ASF- at the time of writing present in the Czech Republic, Germany and Poland and more recently Sweden from our selected cases. This has resulted in an enhanced need for caution when processing carcasses and by-products. ASF control measures are. on paper, harmonized across EU states through guidance documents (http://data.europa.eu/eli/reg_impl/2021/605/oj; https://ec.europa.eu/food/system/files/2020-04/ad_control-measures_asf_wrk-doc-sante-2015-7113.pdf). However, despite particular zoning instructions, outbreaks have been dealt with somewhat heterogeneously (Sauter-Louis *et al*. 2022). In the Czech Republic, following outbreaks in 2017 and 2022, found carcasses must be immediately reported to authorities as part of a passive surveillance scheme and sealed in plastic, while samples are also taken from animals in designated veterinary zones. Five percent of hunted carcasses are also sampled for Classical Swine Fever (CSF).

Following an ongoing outbreak beginning in 2014, Poland has developed a similarly strict ASF monitoring and sanitation regime whereby all carcasses must be tested. Gutting must take place on washable and disinfectable surfaces, or on impermeable, disposable materials in the field; full carcasses, including internal organs, transported in sealed containers or bags; storage facilities equipped with disinfection mats for vehicles and shoes; and carcasses kept in cold storage with access for veterinary services. While no meat can be sold from carcasses obtained in sanitary culling zones, Polish hunters can enlist specially trained persons to butcher other felled animals to sell as meat (Tarasiuk Kand Giżejewski, 2021). Importantly, unaffected European countries have also enacted biosecurity plans in preparedness for outbreaks, either by prohibiting meat imports from ASF-positive countries, such as Norway (in contrast to Sweden which has not), or by investing in campaigns- often targeting hunters- raising awareness about the preventative measures needed to minimize the risk of importing ASF from ASF-positive countries (Hedmark *et al*. 2021).

The relative rigour with which wild boar meat must be processed and tested compared to other game meat- particularly as fears increase around diseases-, complicates its distribution. Everywhere under EU legislation, growing emphasis has been placed on its traceability, with legal requirements to attach identity tags to carcasses through various stages of processing, and labels for meat imports/exports stating it was processed in approved and certified in EU-licensed facilities. In Sweden, for example, it is estimated that only about fifteen percent of potential meat is sold, despite high demand. In 2019, despite harvesting and producing wild boar meat in equal volumes, Sweden imported around 2000 tonnes from Germany, Poland, and Denmark; while Norway, a non-EU country, imported 10–18 tonnes during 2016–2020, with Sweden their dominant supplier.

## 5. Changing socio-legal geographies of wildlife governance

This overview of the socio-legal cultures of wild boar management highlights, first, its heterogeneity across Europe, reflecting the diversity also found across US states (Smith *et al*. 2023). This presents a challenge to organizations, such as ENETWILD who increasingly try to monitor, advise, and promote more integrated, cross-border approaches to European ungulate governance. Although some countries may have similar approaches due to their shared regional histories, such as the Germanic style management clusters (Putman 2011), or else piggyback on one another’s infrastructures, like Norway utilising the Swedish Veterinary Institute for testing samples, others are distinct from one another. Second, we have also seen how there are shared bio- and necropolitical shifts re-shaping management in similar ways. Broadly manifesting through authorities’ attempts to improve wild boar monitoring and regulation, and resulting in intensified culls and outsourcing responsibility to hunters, these shifts appear to have been triggered by three interrelated factors: (i) wild boar (over)abundance and population growth; (ii) biosecurity and disease risk; and (iii) technological innovation.

The first trigger of change are conceptions of (over)abundance and population growth. Across Europe, established monitoring methods have indicated an increase in wild boar numbers, as well as other ungulates, partly due to rural depopulation, evolving agricultural and forestry practices, and warming climates (Linnell *et al*. 2020). Relatedly, declining numbers of hunters and their aging demographic have further affected the effectiveness of long-standing management approaches and their capacity to fulfil authorities’ objectives (Boumendjel *et al*. 2016; Graitson, Barbraud, and Bonnet 2019). Accordingly, several European countries including France and Spain, have now made it easier for new hunters to (i) pass license exams and (ii) lease hunting land to help fulfil cull quotas. Encouraging new hunters and utilizing a carrot-and-stick approach – by penalizing hunters for damage and subsidizing them for kills – is understood by authorities as a decisive strategy to help meet the challenge of wild boar management (Vajas *et al*. 2023).

Moreover, translating animals into numbers by estimating populations, relative abundance and density, reflects a commonplace biopolitical strategy to govern life at large scales (Biermann and Mansfield 2014). In pooling data on wild mammals, ENETWILD and citizen-science initiatives such as Mammalnet hope to facilitate more effective governance. Importantly, this *numero-politics* (Boonman-Berson, Driessen, and Turnhout (2019) helps to determine whether animals exceed perceived ecological and cultural carrying capacities, and become *over-*abundant. Exceeding such thresholds commonly results in more rigorous attempts to contain and cull and, as with wild boar, change management legislation and policy.

Wild boar categorization is framed by various factors including (i) historically established thresholds or ‘cut-offs’ that determine (non-)nativeness; (ii) their origins, whether through anthropogenic (re)introductions or ‘natural’ (re)colonization; (iii) their genetic purity; and (iv) perceptions of appropriate topo-geographical inhabitation, whether wooded, urban, mountain, or agricultural environments. However, it seems the potential speed with which their populations can grow and expand is an increasingly critical qualifier determining legal status, and the bio- or necro-political agendas that determine management. Broadly, wild boar are increasingly framed as “native invader[s]” (Simberloff 2011), or animals perceived as invasive in their historic range. Although Swedish Parliament re-classified once extirpated wild boar as a (re)naturalized ‘native’ species, this is something of an outlier. Arguably, the categories applied to (re)introduced wild boar in Great Britain and Norway – as feral and invasive respectably - were, in part, determined by fears of proliferation. Likewise, their changing categorization in other countries – for example, Poland and Germany – is also influenced by population transformations and growth.

Beyond varying levels of abundance, a second notable trigger are biosecurity crises. While biosecuritization might be understood as an ongoing biopolitical process rather than a one-off event (Epstein, von Essen, and Wilmer 2021), critical health and disease threats transform management (Broz, Arregui, and O'Mahony 2021). Wild boar have long engendered the interest of epidemiologists and concerns of policymakers, as exemplified by *trichinella* policy and past outbreaks of Foot and Mouth Disease and CSF. However, the recent spread of ASF and the threat it presents to the European pork industry has acted as a powerful catalyst for a new, intensifying biosecurity regime. It has prompted a suite of socio-legal changes, with concerns pertaining to wild boar-borne diseases reconfiguring responsibility arrangements and determining how relevant actors should hunt, manage, and process wild boar and their meat, and in some cases that of other game species. Indeed, databases and initiatives from the World Organisation for Animal Health (such as the WAHIS-Wild, World Animal Health Information System) and ENETWILD have been partially driven by pan-European anxieties over ASF (Cardoso *et al*. 2022).

The will to voluntarily help authorities amidst biosecurity crises may differ, as has been found in other European responses to ASF. For example, Latvian hunters internalized surveillance as a duty rather than out of any financial incentives (Sauter-Louis *et al*. 2021); Lithuanian hunters helped when there was sufficient infrastructure in place to do so, and they received feedback on their efforts (Stončiūtė *et al*. 2021); and in Belgium volunteer-hunters worked closely with authorities (Desvaux *et al*. 2021). In Italy, however, responses to a recent ASF outbreak were hindered by the poor relationship and communication between the state, hunters and farmers (Palencia *et al*. 2023b). In the current ASF outbreak in Sweden, hunters and state work collaboratively on the ground with seeming praise from both sides, but a third actor promoting agricultural interests has antagonized its longstanding relationship with hunters by suggesting that ninety percent of the wild boar population in Sweden should now be eradicated. The reasons for these multi-actor responses to crises are complex and numerous, building on past traditions of wildlife management and disease monitoring, and the relative standing of hunting cultures in relation to other societal interests.

The ‘emergency modalit[ies]’ raised by risky diseases emphasize epidemiological logics and their associated practices – surveillance, monitoring, and management through containment, quarantine and separation – within wildlife management infrastructures (Tirado, Enrique, and Moya 2021). Preparedness and responses to ASF, as well as other wild boar related diseases and parasites, have seemingly transformed the temporalities, intensities and scales of traditional wildlife management models. In addition to the aforementioned concerns about (over)abundance, ASF has spurred more rigorous attempts to monitor populations, epidemiological health, and sanitation protocols, even in countries without outbreaks and only modest wild boar populations, such as Great Britain and Norway. This threat has resulted in intensive wild boar culls, elaborate surveillance regimes and regulatory border initiatives, including the erection of wildlife fences along national borders (Emond, Breda, and Denayer 2021).

Biosecuritization is also transforming the circulation of wildlife-related commodities across Europe, such as the trade of meat and hunting tourism. The intention of biosecurity is commonly to separate ‘good’ and ‘bad’ circulations of life, however, tightened border regimes disrupt trade, travel, transportation and tourism (Hinchliffe and Lavau 2013), not by halting them, but through increased intervention (Hinchliffe 2015, 31). Announcing a ban on imports from one country also becomes a political move. These are not merely physical, such as bans on the export/import of wild boar products, but also manifest through ‘soft’ prohibitions and restrictions, as highlighted by ASF guidance relating to transborder travel and hunting best practice. At the same time as restrictions on mobilities of animals, products and people might tighten, managers also contend that collaboration needs to increase across Europe so that, for example, countries that “lack a tradition and knowledge around wild boar management” can supposedly learn from those that do (Hedmark *et al*. 2021, 47). Such a paradoxical protectionist and open, cosmopolitan orientation to wild boar management suggests an uncertain future.

A final trigger for change in management is technological and practical innovation, which has transformed both monitoring and practices of control. This has not only been aided by advances in the technologies available to hunters (from weapons to information communication systems), but also shifts in their legislation and governance, many of which have been prompted by overabundance and biosecurity logics. First, surveillance and monitoring regimes appear increasingly guided by top-down concerns about wild boar risk, with numerous forms of data pooled into central databases that facilitate biopolitical strategies of regulation. At the same time, species monitoring is to use citizen science in the future, such that ‘….scientists are no longer experts translating information about wild animals with the public but act as audiences themselves, receiving information at the same time and in the same way as the non-scientific public’ (Hawkins and Silver 2023, 5). While they have always been important actors in wild boar management, hunters have become more diverse ‘boundary agents’ whose responsibilities and knowledge have multiplied as they are increasingly expected to monitor and regulate disease, as much as population.

Relatedly, hunters’ technological infrastructures and usage has rapidly expanded, and commonly includes trail and thermal imaging cameras, mobile phones, photographs and videos. Increasingly, surveillance occurs through a digitally mediated way of ‘knowing’ wild boar (von Essen *et al.* 2023). Surveillance technology has adapted to nocturnal animal rhythms, with infrared and thermal vision becoming central tools in documenting where and how many wild boar are present. The rise of cameras has not only helped evidence claims around presence or behavior but also potentially elevated the standing of local knowledges, insofar as they may demonstrate the same evidentiary rigour as data put forward by experts. This has meant that management commonly incorporates multiple actors, including civilians who may report observations and personally retrieved data, further diffusing responsibility among non-state actors. Second, technological changes have also facilitated necro-political agendas seeking to control and, in some cases, eradicate wild boar. Improving night vision and thermal imaging technologies have enabled safer shooting at nighttime, with legislative changes enabling practices that have hitherto been commonly banned.

## 6. Concluding thoughts

The maxim ‘one Europe – many management objectives’ for wildlife (Morellet *et al*. 2011) is illustrated in the many different cultural and legal traditions surrounding hunting and wildlife management. At the same time, similarities across Europe become apparent when comparing wild boar management here to other parts of the world. For example, poisons are used against wild pigs in Oceania and North America, but not in Europe (Beasley *et al*. 2021). Further, other countries, such as South Korea, purportedly look to a ‘European approach’ to control ASF (EFSA and Annette Boklund 2018) which involves fencing, zoning and carcass searches.

This paper has shown a sample of such situated management realities with the aim of showing how wild boar fit into and affect them. In wild boar management, some grand narratives of biosecurity now ostensibly look to be homogenizing management practices across the continent, which center on surveillance, control, and sanitation. In practice, however, such initiatives are nationally and regionally re-interpreted. Wild boar management, in part, becomes a question of ‘whom can we learn from?’ among European neighboring countries. However, for most of the countries reviewed above, wild boar management appears to constantly be finding its footing and is always in the process of change. In some ways, the wild boar has, and may further become, a training wheel for European wildlife management and hunting practices, appearing as it does to be the first port for new technologies, new hygiene requirements and even new kinds of hunters.

## Notes

1 ERC consolidator grant entitled “Veterinarization of Europe? Hunting for wild boar futures in the time of African Swine Fever”.

2 These countries were selected primarily as they correspond to the research locations of members working on the ERC project. However, they also represent an interesting cross-section of management approaches as identified by Putman (2011).
